# A Comparison of Inductive Sensors in the Characterization of Partial Discharges and Electrical Noise Using the Chromatic Technique

**DOI:** 10.3390/s18041021

**Published:** 2018-03-29

**Authors:** Jorge Alfredo Ardila-Rey, Johny Montaña, Bruno Albuquerque de Castro, Roger Schurch, José Alfredo Covolan Ulson, Firdaus Muhammad-Sukki, Nurul Aini Bani

**Affiliations:** 1 Department of Electrical Engineering, Universidad Técnica Federico Santa María, Av. Vicuña Mackenna 3939, Santiago de Chile 8940000, Chile; 2 Department of Electrical Engineering, Universidad Técnica Federico Santa María, Av. España 1680, Valparaiso 2340000, Chile; johny.montana@usm.cl (J.M.); roger.schurch@usm.cl (R.S.); 3 Department of Electrical Engineering, São Paulo State University, Av. Eng. Luiz Edmundo Carrijo Coube 14-01, Bauru 17033-360, Brazil; bruno.castro@unesp.br (B.A.d.C.); alfredo.ulson@unesp.br (J.A.C.U.); 4 School of Engineering, Robert Gordon University, Garthdee Road, Aberdeen AB10 7GJ, UK; f.b.muhammad-sukki@rgu.ac.uk; 5 UTM Razak School of Engineering and Advanced Technology, Universiti Teknologi Malaysia, Kuala Lumpur 54100, Malaysia; nurulaini.kl@utm.my

**Keywords:** partial discharges (PDs), inductive sensors, chromatic technique, condition monitoring, electrical insulation condition

## Abstract

Partial discharges (PDs) are one of the most important classes of ageing processes that occur within electrical insulation. PD detection is a standardized technique to qualify the state of the insulation in electric assets such as machines and power cables. Generally, the classical phase-resolved partial discharge (PRPD) patterns are used to perform the identification of the type of PD source when they are related to a specific degradation process and when the electrical noise level is low compared to the magnitudes of the PD signals. However, in practical applications such as measurements carried out in the field or in industrial environments, several PD sources and large noise signals are usually present simultaneously. In this study, three different inductive sensors have been used to evaluate and compare their performance in the detection and separation of multiple PD sources by applying the chromatic technique to each of the measured signals.

## 1. Introduction

Under normal operating conditions, insulation systems of high voltage electrical equipment are constantly subjected to multiple mechanical, electrical, thermal, and environmental stresses [1,2]. Although these stresses do not produce immediate failures on insulation, over time, a slow but effective aging of the material is initiated, due to the appearance of a series of degradation processes such as decomposition, erosion, oxidation, hydrolysis, chemical attack, mechanical creep, tracking, treeing, cracking, pitting, or dry-band arcing, among others, eventually leading to the loss of the insulating properties of the material until, finally, complete electric asset failure occurs [3,4,5]. In pre-failure stages, it is common to find small displacements of charge or ionization phenomena of very low energy and short duration inside the insulation or on the insulation surface. These ionizations are known as PD and can be considered as an important indicator when characterizing the state of the insulation of a machine or equipment [6,7,8,9]. According to their nature, PD can be divided into three types: internal, superficial, and corona; this distinction is important since not all sources of PD are equally harmful to electrical equipment. As documented in numerous studies [7,10,11], each PD type can contribute to the degradation of an insulation system in different ways. However, internal PDs are considered the most harmful sources, since they occur inside the insulation and, if not detected in time, irreversible damage can lead to catastrophic failures.

In this context, the development of PD diagnosis has received an increasing amount of attention in recent decades so that unexpected failures in high voltage equipment might be avoided. Both industry and science seek to develop monitoring systems that guarantee reliable operation and, at the same time, reduced maintenance costs. Therefore, PD measurement systems must be capable of detecting all types of PD sources; otherwise, measurement may not yield correct diagnoses of power equipment.

Traditionally, the identification of the PD source type is carried out through so-called phase-resolved partial discharge (PRPD) patterns [12,13]. These patterns represent the PD activity with the applied voltage, where all the PD sources can exhibit a specific PRPD pattern that makes it clearly identifiable. Unfortunately, in practical applications, such as measurements made in the field or in industrial environments, it is very difficult to find or capture a single type of source, so identification cannot be achieved directly from the PRPD patterns acquired during the test. This happens because the PRPD patterns are usually complex and can be formed by more than one type of source acting simultaneously, in addition to the presence of pulses of great amplitude without phase correlation associated with electrical noise, which can hide or mask other sources [14]. As a consequence, the identification of PD sources through the PRPD plots under these circumstances becomes practically impossible for an operator or any intelligent identification system.

Regarding PD source separation, several studies have indicated [15,16,17] that, in order to improve the identification process, it is necessary to incorporate a separation step that allows different PD sources to be properly separated and grouped, so that the sources can be analyzed individually and displayed in their respective PRPD pattern. This separation step can be taken by dividing the complex PRPD pattern into different sub-patterns, where each is associated with a specific PD source. The above procedure can be implemented in a more effective way if high bandwidth instrumentation systems based on sensors that can be easily coupled with standard detection circuits are used [18,19,20]. The main advantage of using this type of sensor is that they allow for the capture of all of the information of the signal, which is used to identify a number of characteristic parameters (in temporal and frequency domain) for the sources obtained, which may also serve for the subsequent separation step [21].

In this sense, the chromatic technique, presented initially in [22], can generate different signatures for all types of PD by calculating three different parameters: signal energy (*E_b_*), characteristic angular frequency (*ω_c_*), and signal equivalent bandwidth (*B*). Other studies based on this technique have allowed for characterization in a separation map, radio frequency emissions from surface PD sources, internal PD, and noise, which were captured with a log-periodic antenna [23]. However, this study had its limitations. First, due to the type of antenna used, it was not possible to characterize corona activity. Second, as they were signals from antennas (radiated signals), the signal-to-noise ratio (SNR) was high, which reduced the sensitivity of parameters used in the characterization of the sources. Third, multiple PD sources were not characterized.

In this work, three inductive sensors commonly used in industrial and laboratory measurements have been used: a high-frequency current transformer (HFCT), an inductive loop sensor (ILS), and a Rogowski coil (RC). Their separation ability was evaluated and compared by the application of the chromatic technique. This was carried out by calculating the values of *E_b_*, *ω_c_*, and *B* for the PD signals obtained from the sensors and determining the discrimination capability of those parameters. As corona, surface, and internal PDs are common in power transformers, these types of PD sources were selected in order to produce similar signals to those produced in practical applications.

This paper is divided into six sections. Section 2 and Section 3 present the basic concepts of the inductive sensors and the chromatic technique approach, respectively. The experimental setup is described in Section 4, and the results are discussed in Section 5. Finally, the conclusions of this work are presented in Section 6.

## 2. Inductive Sensors for the Detection of PD

For the measurement of PD, different types of inductive sensors have been implemented [14,16,21,24,25]. One of the main advantages of these sensors is that they do not require galvanic contact with the system or equipment under test, so the acquisition procedure is carried out without altering the measurement circuit. In general, these sensors can have bandwidths of the order of MHz (depending on the materials used and their construction principle), which allows them to be an adequate option when capturing all the information in time and in frequency of those signals associated with PD or electrical noise. 

**Figure 1:**
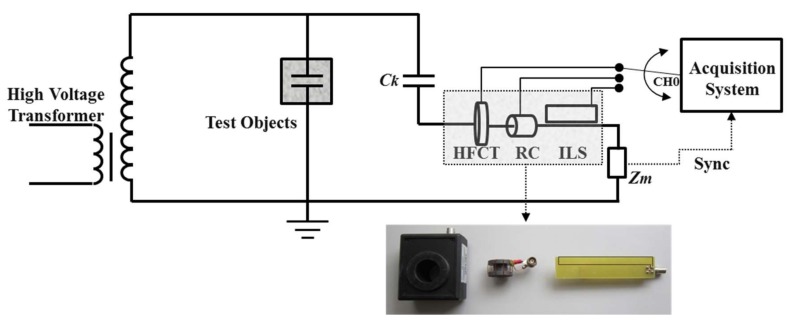
Experimental setup and location of the three sensors to record the partial discharge (PD) signals.

The experiments described in this paper used a commercial HFCT, an ILS, and an RC. These sensors were selected since their behavior and performance were characterized in previous works [21,23,24,25,26]. In addition, the RC and ILS are easy to manufacture and are inexpensive, which makes them widely used in industry and laboratory measurements [23,24,25,26]. The following are the most important characteristics of these sensors:

- **HFCT:** A commercial sensor Clamp High-Frequency Current Transformer (model 30 mm of the company *Techimp-System*) was used. This ferromagnetic core sensor has a sensitivity of about 25 dB and a bandwidth between 1 and 80 MHz [21]. Its dimensions are 72 × 97 × 35 mm, the central hole being where the line that leads the pulses of the PD and the electrical noise of 30 mm is located (see Figure 1).
- **ILS:** The prototype used is the same one described in [21], which presents a derivative behavior between 0 and 34.69 MHz, where it reaches a sensitivity of 17.5 dB. As expected, the sensitivity of this sensor is much lower than the one obtained for the HFCT; this is due to its constructive principle and the absence of ferromagnetic material, which increases the concentration of magnetic flux.
- **RC:** As indicated in [24], this prototype has a sensitivity of approximately 12 dB; its behavior is derivative between 0 and 9 MHz; however, for bands 9–60 MHz, the sensor output is proportional to the measured signal. Among the three sensors, the RC has the lowest sensitivity.

## 3. The Clustering Process Based on a Chromatic Technique Approach

This methodology provides a series of parameters that facilitate the extraction of information from a complex group of signals whose characteristics cannot be easily identified. It has been demonstrated that this technique also enables, for some types of applications, the identification of the correlation of the parameters that define the signals, determining if a group of signals can be identifiable from a chromatic point of view [22,23,27,28,29].

According to this technique, the signal processing for their classification is carried out by means of signal principal parameters. These parameters include energy content, average frequency, average time, RMS bandwidth, and RMS duration. In this work, three of them are used in order to classify the signals: signal energy (*E_b_*), characteristic angular frequency (*ω_c_*), and signal equivalent bandwidth (*B*). These parameters are described below.

Signal energy content (*E_b_*) can be expressed as follows:

(1) $$E_{b}=\int {\left|f\left(t\right)\right|}^{2}dt=\frac{1}{2\pi }\int {\left|f\left(\omega \right)\right|}^{2}d\omega$$

Average frequency or characteristic angular frequency (*ω_c_*) can be expressed as follows:

(2) $$\omega_{c}=\frac{\int \omega {\left|F\left(\omega \right)\right|}^{2}d\omega }{2\pi E_{b}}$$

Equivalent bandwidth (or RMS bandwidth, *B*) can be expressed as follows:

(3) $$B\;=\sqrt{\frac{1}{E_{b}}\int {\left(\omega -\omega_{c}\right)}^{2}{\left|F\left(\omega \right)\right|}^{2}d\omega }$$

where F(ω) is the Fourier transform of f(t), ω is the angular frequency, and *t* is the time.

## 4. Experimental Setup

An NI-PXI-5124 system with a 200 MS/s sampling rate, 12 bits of vertical resolution and 150 MHz of bandwidth was used for the acquisition, processing, and visualization of the data obtained from PD activity and electrical noise.

According to what is shown in Figure 1, the information captured by the three sensors was sent sequentially to one of the channels that has the PXI acquisition system (Channel 0). The measurement procedure was carried out sequentially, since the system does not have three channels required for a simultaneous connection of the three sensors. For this reason, once a stable PD activity was ensured on the test object, each sensor was sequentially connected in the order HFCT, RC, and ILS. Additionally, the synchronism signal (to the applied voltage frequency) was taken from the measurement impedance *Z_m_*, in order to establish the phase of occurrence of the pulses with respect of the applied voltage. The ambient conditions at the beginning of the tests were a temperature of 29 °C, a relative humidity of 43%, and an atmospheric pressure of 1019 mbar. These conditions did not change significantly during the tests. The remaining part of the indirect measurement circuit used for the measurement process, which is according to the IEC 60270 standard [12], is described below:

- a 10 kVA transformer that provides up to 100 kV to apply high voltage on each test object.
- a coupling capacitor *C_k_* of 1 nF used as a low impedance path for high frequency currents. In this way, the PD pulses cross the line where the three sensors described in Section 2 are connected.
- Test objects:
  - *a point–plane experimental test object (corona discharges)*: A tip connected to high voltage was placed 5.5 cm above a grounded metal plane, obtaining the stable activity of corona PD. Although the voltage levels applied to this test object in all experiments were completely controlled, a thin layer of insulation was placed over the ground plane to avoid any possible damage to the acquisition system, in case of a total breakdown of the air gap between the tip and the plane.
  - *ceramic bushing (surface discharges)*: This test object consisted of a bushing insulator made of porcelain, commonly used in medium-voltage transformers. Additionally, this sample was surface-contaminated with a 40% saline solution, in order to obtain PD on its surface. A stable partial surface discharge activity was achieved applying high voltage between the ends of the bushing through two cylindrical steel electrodes.
  - *sheets of NOMEX paper with cavity (internal discharges)*: For this test object, nine sheets of NOMEX paper were stacked together, vacuum packed, and immersed in transformer mineral oil between two electrodes subjected to high voltage. The three central sheets were previously perforated with a needle 0.5 mm in diameter in order to obtain a cylindrical vacuole that provided internal PD.

These test objects were selected in order to obtain PD sources similar to those produced in a transformer and to evaluate the individual behavior of each PD source type when the chromatic technique was applied to the signals recorded for each sensor.

The acquisition of the PD pulses was carried out in intervals of 20 ms in order to obtain the data for an entire cycle of applied voltage. After acquisition, the PD or electrical noise pulses were separated into time windows of 1 or 4 μs, so an individual analysis was guaranteed for all measured pulses.

After the measurements over the laboratory test samples were carried out, an oil-paper insulated distribution transformer (300 kVA, 12/0.42 kV), affected by the internal PD in one of its windings was used as an object to test the chromatic separation technique. Simultaneously, the point–plane configuration was connected in parallel in order to obtain corona PD for the same voltage level. Naturally, the acquired PD signal had an additional source corresponding to the electrical noise present in the laboratory if a low trigger level was kept.

In the experimental measurements where only a single PD source was present, approximately 600 pulses were acquired. For the case where more than two sources were active, about 1500 pulses were stored in order to obtain a more significant amount of data from a statistical point of view.

## 5. Measurements and Experimental Results

### 5.1. Electrical Noise Characterization

For the test objects described in Section 4, the acquisition of electrical noise in a shielded high-voltage laboratory was carried out by applying a low trigger level in the PD acquisition system and a low voltage (1 kV), to guarantee the sync signal needed to build the PRPD plots but without PD activity. This procedure exclusively measured the electrical noise of the environment in the laboratory.

Trigger levels were established individually for each sample according to the SNR of each sensor, in order to capture the maximum noise levels (see Table 1). When a lower trigger level was used, the acquisition system tended to saturate due to the large number of pulses that can reach the measurement channel.

**Figure 2:**
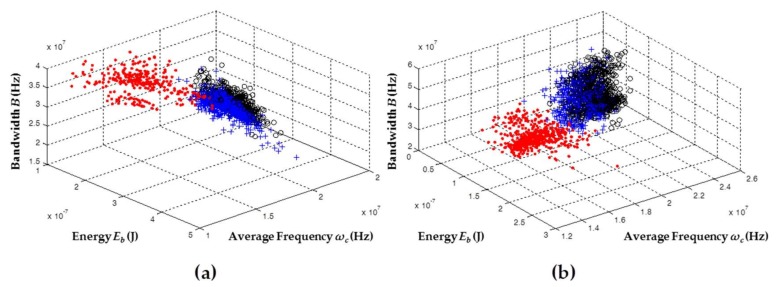

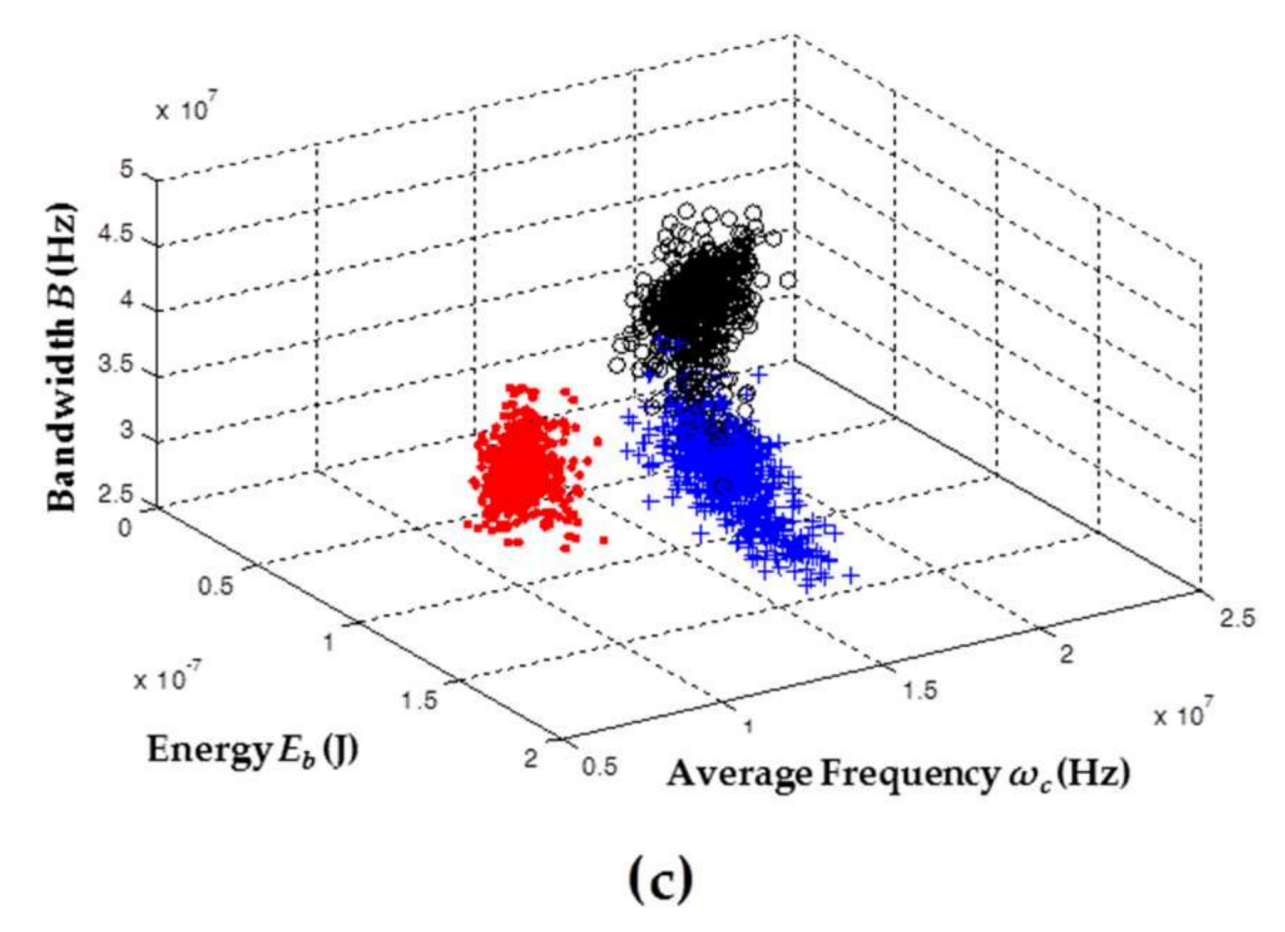
Representation of clusters associated with electrical noise for each sensor: (**a**) high-frequency current transformer (HFCT); (**b**) inductive loop sensor (ILS); and (**c**) Rogowski coil (RC). Red dots: point–plane; black circles: ceramic bushing; blue crosses: NOMEX.

Figure 2 shows the results obtained by applying the chromatic technique whereby the noise signals recorded in the different test objects are clustered and separated. Although the measurements were made individually at different times, the results for the three test objects are represented in the same map, in order to jointly analyze the behavior of the electrical noise captured in the different test objects for each sensor.

Figure 2a shows the noise clusters that were obtained with the data recorded with the HFCT sensor. In this figure, there is a clear overlap between clusters associated with electrical noise in ceramic bushing (black circles) and sheets of NOMEX paper (blue crosses). However, the cluster of electrical noise in the point–plane configuration (red dots) had a displacement in the *ω_c_* axis due to a lower energy in the noise pulses recorded for this test object. Analyzing the signals measured with the other two sensors, a similar behavior was also observed in each cluster. For both ILS (Figure 2b) and RC (Figure 2c), the cluster associated with the electrical noise signals in the point–plane configuration continued to maintain a low energy level, which caused this cluster to shift on the *ω_c_* axis with respect to the other clusters. Like the signals captured with HFCT, the ILS concentrated in the same zone the pulses of noise captured in the ceramic bushing and the insulation papers; however, for the RC, a displacement in the equivalent bandwidth (*B*) of these sources was observed, which caused them to be located in different areas, leaving the clusters in different zones.

In general, although it was the same source of noise, this variation in the position of the clusters can be related to changes in the equivalent capacitance of the test object, the type of sensor (depending on their frequency response and gain), and the total path traveled by the signals, all of which imposed different spectral and temporal behavior to the signal when it reached the point where the sensor was connected [16,21]. This approach explains why the same source of electrical noise captured by each sensor resulted in a different location in the 3D map.

Figure 2 shows that the highest energy levels (*E_b_*) were recorded by HFCT (up to 4 × 10^−7^ J), followed by ILS (up to 2 × 10^−7^ J) and then by RC (up to 1.5 × 10^−7^ J). This behavior was in agreement with the gain levels of the sensors shown in Section 2, the highest being from the HFCT (25 dB) and the lowest being from the RC (12 dB).

### 5.2. Partial Discharge Characterization

The PD activity was characterized in the test objects by applying a trigger level that was higher than the noise level measured in the previous section. Subsequently, the voltage level was increased until a stable PD activity was obtained. This measurement procedure guaranteed that only the pulses associated with PD were captured in each experiment.

**Figure 3:**
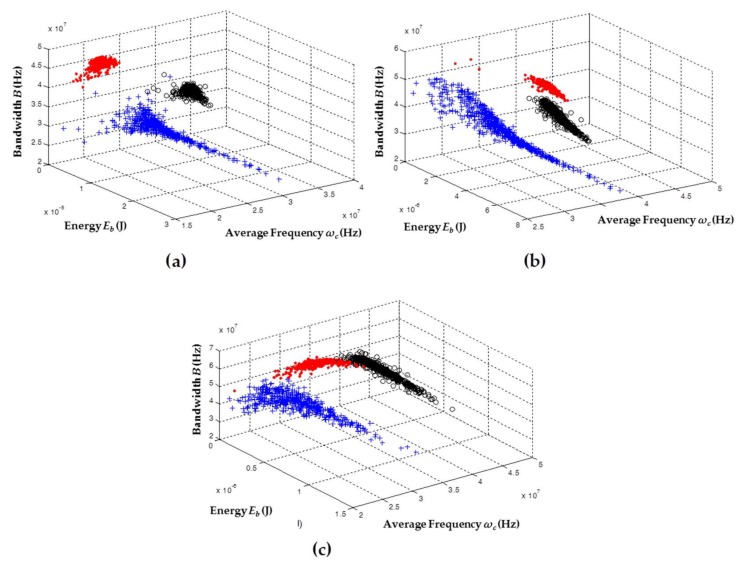
Representation of the clusters associated with PD for the three sensors: (**a**) HFCT; (**b**) ILS; and (**c**) RC. Red dots: point–plane; black circles: ceramic bushing; blue crosses: NOMEX.

Table 2 shows the trigger and voltage levels used for these experiments. The results obtained in terms of clustering are shown in Figure 3. Once again, for each sensor, the clusters are displayed in a single map so that the behavior of each sensor for different PD sources can be analyzed.

As shown in Figure 3, the signals captured with the sensors, for each one of the PD sources, took different positions and forms in the 3D classification maps of the chromatic technique. This validated that all types of PD sources analyzed here can exhibit a different behavior in function of the *ω_c_*, *B*, and *E_b_* parameters, facilitating the differentiation and separation of PD sources, even in the cases where these sources act simultaneously during the process of acquisition. For the data captured with the HFCT (Figure 3a) and the ILS (Figure 3b), the separation between the clusters was much more noticeable than for the data obtained with the RC (Figure 3c), since for this sensor a slight overlap between clusters associated with PD corona (red dots) and surface PD (black circles) was observed.

In terms of dispersion, when comparing the three clusters obtained by the sensors, the cluster associated with internal PD (blue crosses) had the largest variability in the axis associated with the energy of the signals *E_b_*, this variability being more noticeable for the ILS sensor (Figure 3b). Moreover, it was observed that, according to the characteristics of the sensors in terms of gain, the signals captured with the HFCT sensor had higher energy levels (up to 2.5 × 10^−5^ J) than those signals obtained with the ILS sensor (up to 7.3 × 10^−6^ J) and the RC sensor (up to 1 × 10^−6^ J).

It should be noted that, for all sensors, the shape and location of the clusters associated with the same source will not always be the same if any parameter of the measurement circuit, including the equivalent capacitance of the test object or the type of sensor, varies during the acquisition. For example, for the test object that created internal PD (sheets of NOMEX with cylindrical vacuole), when the size of the vacuole increased or decreased, or when the number of sheets or the type of isolation were changed, the cluster associated with this type of source had a different form and position in the map. This behavior has been documented in our previous work [16], where the separation and clustering techniques were based on the extraction of information from the spectral and temporal content of the measured signals of PD and electrical noise. The variability in clusters due to possible changes in the test objects or the measurement circuit itself confirmed that it is not possible to perform source identification by simply evaluating the position or shape of a cluster in the separation map. However, this situation does not affect the main contribution of this article, which is based on clustering and separating sources of partial discharge by applying the chromatic technique, in order to improve the subsequent identification process, either by an expert or by an intelligent identification system.

**Figure 4:**
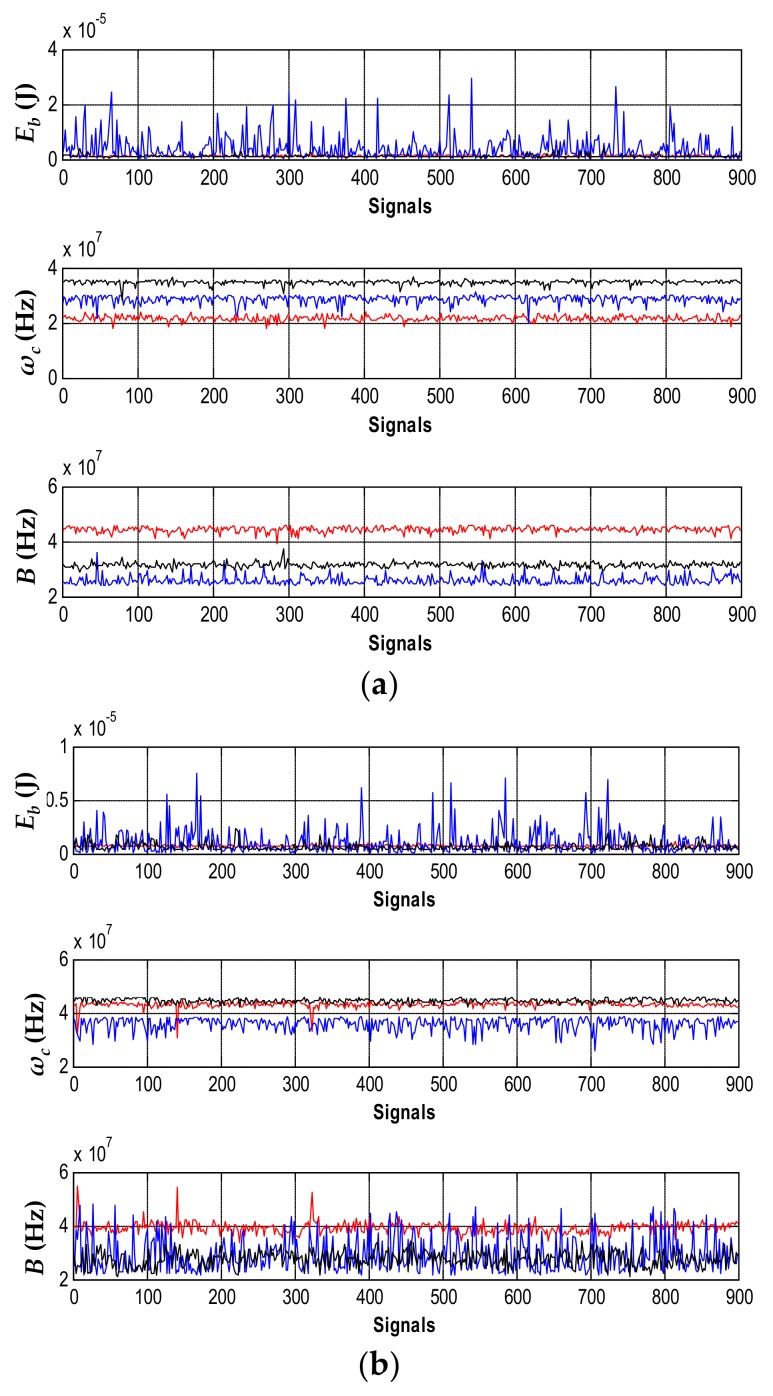

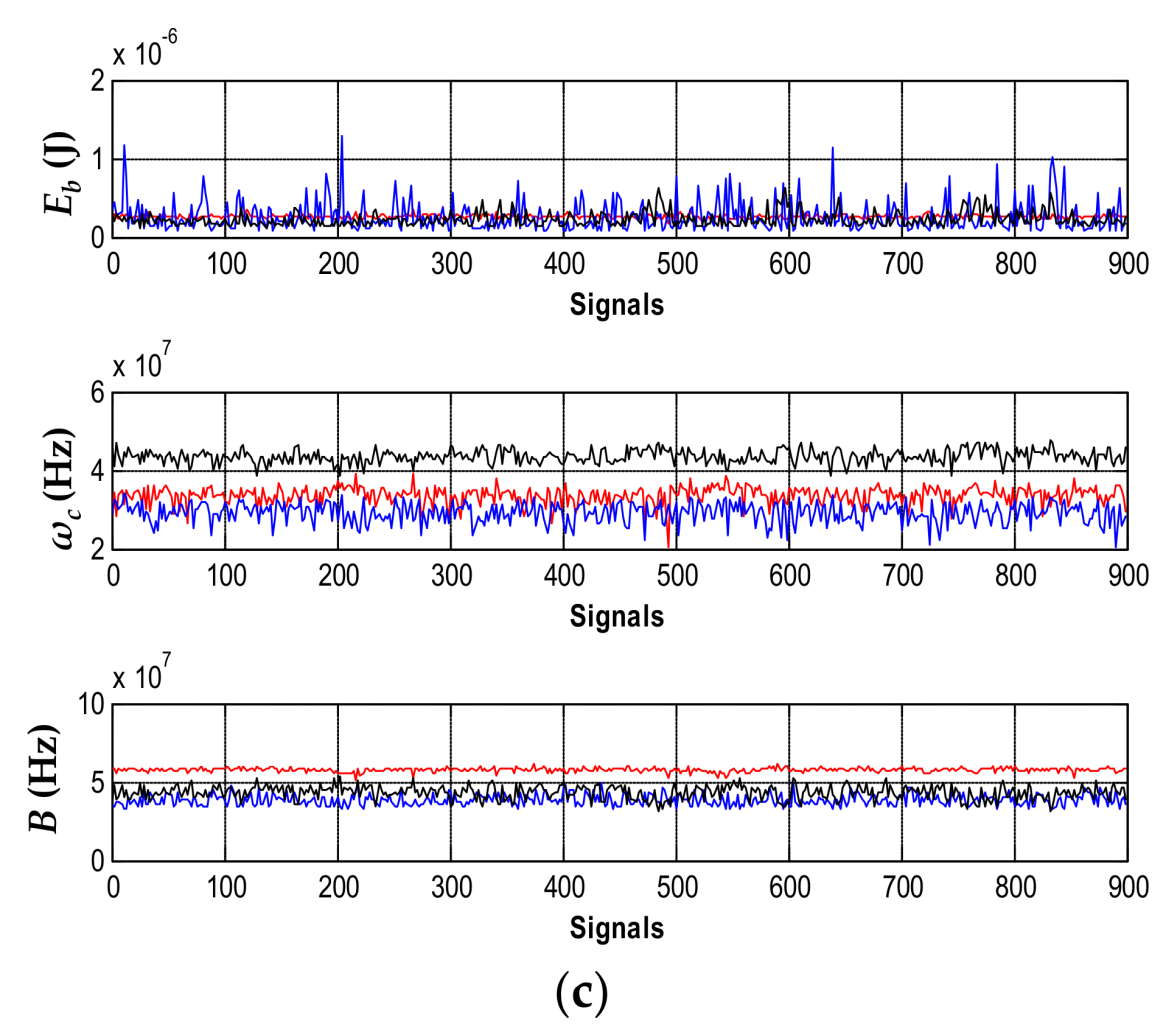
Parameters of the chromatic technique for each of the sensors for the recorded signals: (**a**) HFCT; (**b**) ILS; and (**c**) RC. Corona PD (red), internal PD (blue), and surface PD (black).

*Evaluation of ω_c_*, *B*, *and E_b_ parameters for different types of PD*: In order to evaluate which of the three parameters used in the chromatic technique (*ω_c_*, *B*, and *E_b_*) allowed a better characterization of the type of PD source, the value of these parameters was obtained for all signals recorded with the three sensors in each of the PD measurements. The results are shown in Figure 4.

In all the parameters, PD sources were represented with the same colors of Figure 3, aiming to identify the degree of similarity and/or variability that each parameter had in relation to a specific PD source type.

Figure 4a shows that the parameters *ω_c_* and *B* in the HFCT were those that best characterized the type of PD source, besides presenting a lower variability in the values obtained in all PD pulses (by obtaining low dispersion values in the parameters of the separation map, less dispersed clusters can be achieved, facilitating the selection of the cluster when the representation of its PRPD is required for the subsequent identification process). For the case of the parameter *E_b_*, there was no clear difference between the three types of PD sources.

A similar trend in the three parameters for ILS (Figure 4b) and RC (Figure 4c) was also obtained, where *E_b_* was the least representative parameter when the PD source was differentiated (in addition to having the largest dispersion). Thus, the parameters *ω_c_* and *B* resulted to be better suited for the characterization of PD source. 

**Figure 5:**
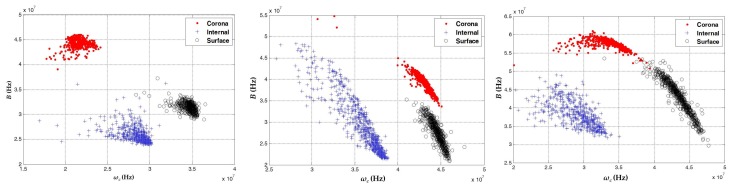
2D representation (*ω_c_* − *B*) of the clusters associated with PD activity for each of the sensors. HFCT (**left**); ILS (**middle**), and RC (**right**).

In order to confirm that *ω_c_* and *B* are sufficient for performing a correct separation among PD sources, the same results shown previously in Figure 3 are depicted in a two-dimensional (2D) map comprising only the variables *ω_c_* and *B*. Figure 5 shows the new 2D maps obtained.

In these new 2D maps, it can also be clearly observed that different PD sources take different positions, which indicates the presence of three different types of sources. 

**Figure 6:**
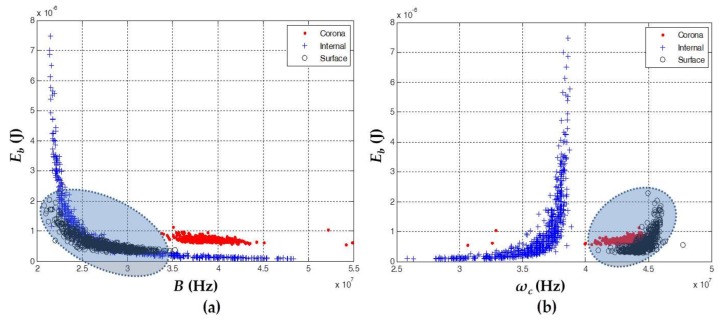
2D Representation of the clusters associated with PD activity for the ILS sensor. (**a**) *E_b_* − *B* and (**b**) *E_b_* − *ω_c_*. The highlighted areas point out the overlapping when the energy (*E_b_*) is used as variable in the maps.

Two different classification options are represented in Figure 6: in (a) the 2D map *E_b_* − *B* and in (b) the *E_b_* − *ω_c_*. The maps shown in this figure correspond to the data taken with the ILS sensor (similar results were obtained with the RC and HFCT sensors). As expected, when the energy was used as a variable in the separation map, an overlapping occurred that did not allow for a proper separation of the different types of sources. This is evidenced in both maps: in Figure 6a, the clusters associated with internal and surface PD are overlapped; a similar situation occurred for clusters associated with corona and surface PD, as shown in Figure 6b. For the case of the signals captured with the other two sensors, overlap was also found. As a conclusion, *E_b_* was not a useful parameter for the clustering process and therefore could be discarded in the analysis.

### 5.3. Partial Discharge Source and Noise Characterization

In the results shown in the previous section, since the data were not taken simultaneously in the same acquisition, it cannot be considered as a good indicator that the chromatic technique serves to separate multiple sources acting at the same time into clusters. Accordingly, it was necessary to carry out measurements of PD sources and/or electrical noise acting simultaneously to validate the technique of clustering multiple PD sources. In this section, through the simultaneous acquisition of multiple PD sources and electrical noise in environments and test objects not so controlled, attempts to evaluate whether or not the *ω_c_* and B parameters are indeed sufficient to make a correct separation and, on the contrary, the energy of the signals becomes useful at separation are presented.

**Figure 7:**
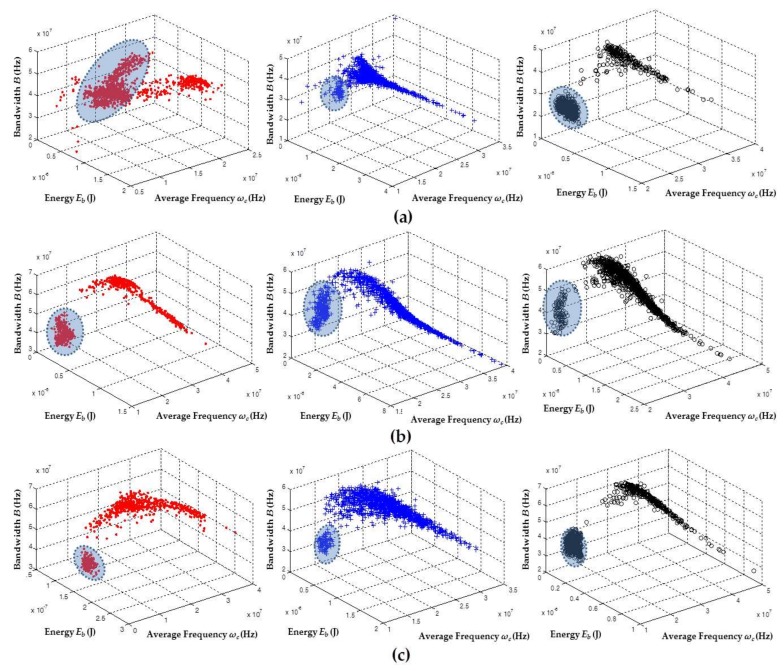
Representation of the clusters associated with PD activity and electrical noise for the three sensors using *ω_c_*, *B*, and *E_b_* parameters: (**a**) HFCT; (**b**) ILS; and (**c**) RC. Corona+noise (red), internal+noise (blue), and surface+noise (black). The highlighted clusters are associated with electrical noise.

In order to simultaneously obtain sources of PD and electrical noise for the test objects, the voltage levels of Table 2 were applied, while the trigger levels shown in Table 1 were maintained in the sensors. Following this procedure during acquisition, the PXI acquisition system was forced to store sources of noise and PD simultaneously. It is worth mentioning that, during the experiment, it was necessary to keep an adequate trigger level to avoid the saturation of the acquisition channel with sources of noise, which prevented the acquisition of higher amplitude sources associated with PD. The results obtained for these experiments are shown in Figure 7.

**Figure 8:**
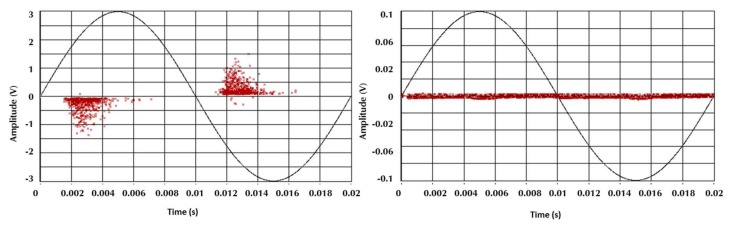
Phase-resolved partial discharge (PRPD) patterns of surface PD (**left**) and electrical noise (**right**) for the HFCT sensor.

Figure 7 shows that the clusters associated with electrical noise (highlighted clusters) took different positions with respect to the clusters of PD. These results confirmed that the technique allows for the effective separation of the sources of PD from the sources associated with electrical noise. Likewise, when clusters corresponding to electrical noise in each of the separation maps were evaluated, a much lower dispersion was observed for this type of source compared to the dispersion obtained for clusters associated with PD. This high dispersion in the PD cluster was much more noticeable in the *E_b_* axis, which coincides with the results obtained in Section 5.2, where the largest dispersion in the data recorded for the PD sources corresponded to the same variable. The behavior in the *E_b_* dispersion for the PD pulses can be explained by analyzing the PRPD patterns of this type of source. For example, if the two PRPD patterns corresponding to the clusters of Figure 7a (right) (surface PD and noise) captured with the HFCT are presented, it can be seen that the variability in the pulse amplitudes associated with surface PD was considerable larger than the variability of the electrical noise (signals without phase correlation), as shown in Figure 8. For this reason, when there is a high variability in the value of the amplitudes of PD signals, the energy associated with these pulses also varies proportionally (see Equation (1)).

For the case of the noise captured with the HFCT shown in Figure 7a (left), it can be inferred that the low variability in noise clusters was not completely fulfilled. However, it should be noted that the gain and bandwidth of this sensor is larger than the other two sensors (see Section 2). For this reason, it is likely that, during the experiment, the HFCT sensor captured additional sources of electrical noise from surrounding laboratories.

In general, when the behavior that the sensors presented using the chromatic technique for separating the PD sources and electrical noise was analyzed, it was clear that there was an adequate characterization of the two source types. For the case of corona PD and electrical noise, the HFCT sensor obtained the lowest separation of the three sensors. When the case of internal PD and noise was evaluated, the separation was similar for the three sensors but was slightly greater for the RC sensor. Finally, for the case of surface PD and electrical noise, it can be considered that the separation was notorious for all sensors since there were few diffuse discharges (signals that can contain spectral components of noise and PD), and the two clusters were more clearly separated from each other. On the other hand, when the position of the clusters of PD in Figure 7 was compared with the position of the clusters that were obtained individually in Section 5.2 (see Figure 3), it can be inferred that the PD sources take similar positions in the separation map, which is expected assuming that there were no significant changes in the test objects and the measurement circuit did not suffer any significant variation in the equivalent capacitance.

**Figure 9:**
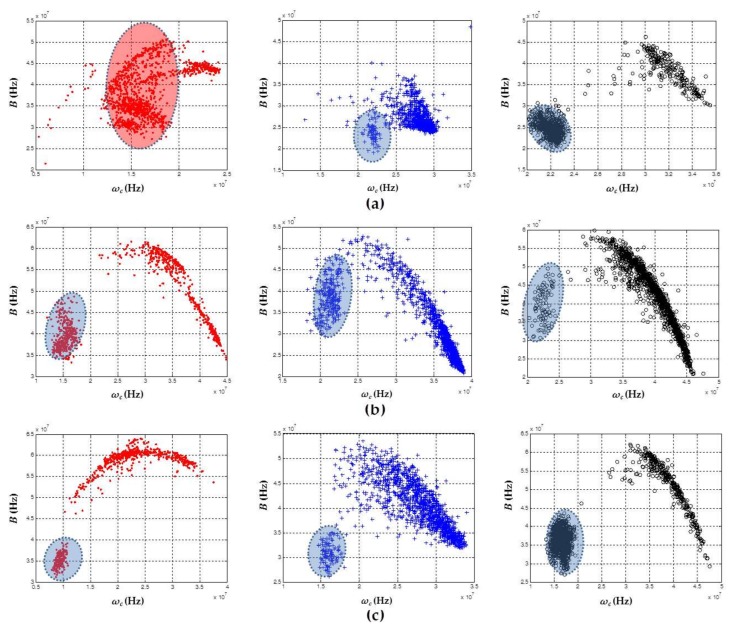
Representation of the clusters associated with PD activity and electrical noise for the three sensors using *ω_c_* and *B* parameters: (**a**) HFCT; (**b**) ILS; and (**c**) RC. Corona+noise (red), internal+noise (blue), and surface+noise (black). The highlighted clusters are associated with electrical noise.

In Figure 9, the separation maps can be observed, but in this case the parameter associated with the energy of the signals (*E_b_*) is not considered. As in the previous section, using the two parameters *ω_c_* and *B*, it was possible to identify the two sources associated with PD and electrical noise in a suitable way for each sensor. 

### 5.4. Partial Discharges in a Distribution Transformer, Corona and Electrical Noise Acting Simultaneously

In this section, the separation capacity of the chromatic technique in an electric asset was evaluated. For this purpose, an oil-paper insulated three-phase transformer (300 kVA, 12/0.42 kV) was used as test object, which had a stable internal PD activity when it was subjected to a voltage of 11.5 kV. In addition, the point–plane configuration was adjusted with a 5 cm gap distance between the point and the plane, and it was placed in parallel to the transformer, in order to simultaneously obtain the corona PD. During the experiment, the trigger level in the acquisition system was set low, in order to obtain a third source associated with electrical noise. This procedure guaranteed the simultaneous presence of at least three different sources: the internal PD in the transformer, the corona PD, and the electrical noise.

Finally, for these experiments, part of the shielding that was located on the walls of the laboratory, which helps minimize the effect of external noise sources from other laboratories, was removed in order to generate an environment similar to that found in a field condition, where different signals (of large amplitude and of broad spectral content) can be coupled to the measurement line.

**Figure 10:**
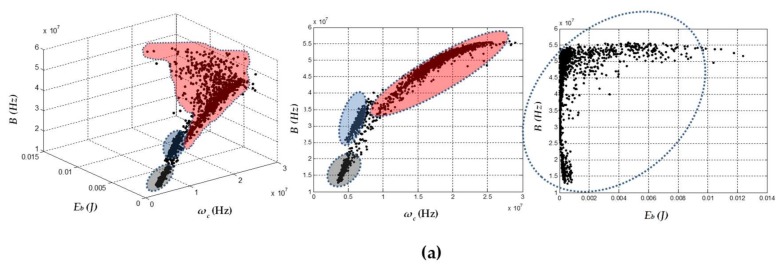

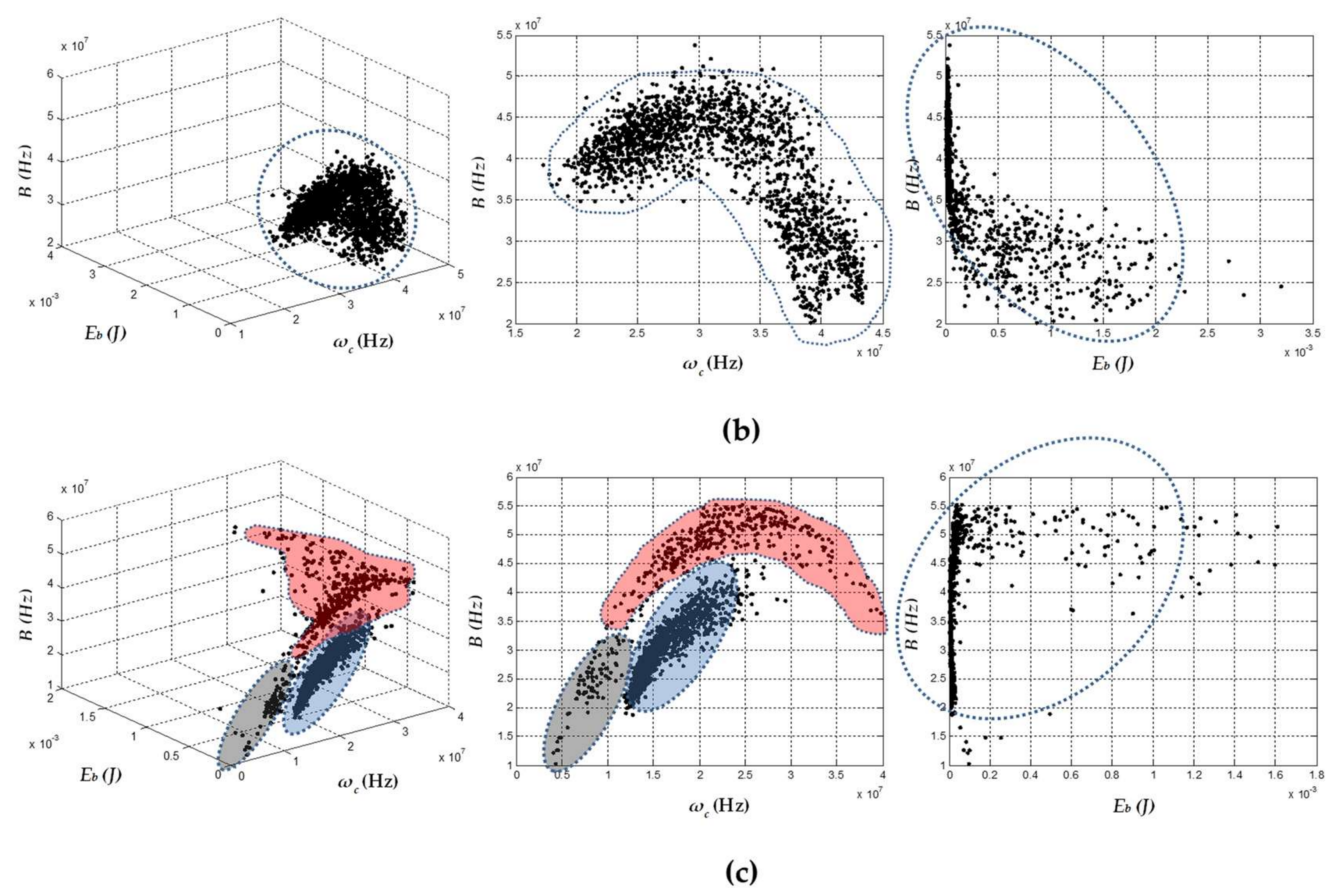
3D and 2D maps obtained for simultaneous PD sources: internal PD in the transformer, corona PD and electrical noise. (**a**) HFCT; (**b**) ILS; and (**c**) RC. The clusters associated with electrical noise were shaded in red, the clusters associated with corona PD in gray, and the clusters associated with internal PD in blue. Those clusters that could not be characterized are without color.

The results obtained for each sensor, using two and three separation parameters are shown in Figure 10. The data recorded with the HFCT and the RC were those that better identified the presence of more than one type of source, although not with the same definition as in the previous experiments, where the test objects were laboratory-prepared and the noise level was lower. Figure 10a,c (left) show that it was possible to characterize the three types of expected sources using the three parameters of the chromatic technique, the clustering ability being more noticeable for the sensor HFCT.

For the 2D maps formed with the parameters *B* and *ω_c_* (Figure 10a,c (middle)), the presence of the three types of sources was also more noticeable for the HFCT sensor. In the case of the RC sensor, the cloud of points associated with electrical noise and corona PD were scattered and tended to overlap at the edge. When many more pulses were recorded during the acquisition, a single cluster could be formed, which would not allow for correct identification of the three types of sources. For the data recorded with the ILS (see Figure 10b), it was not possible to identify in any of the maps a separation or characterization of the PD sources.

As these results show, a more hostile environment, with more noise sources and when using a more complex test object, tends to disturb the signals that the sensors provide to the acquisition system.

One point to consider is that the ILS and RC sensors used in this work did not present any shielding protection. For this reason, they were susceptible to the capture of unwanted signals that could be coupled at any point of the sensor. This phenomenon has already been documented in [24], where the PD signals provided by sensors of this type in some cases had high additional spectral content, associated with external signals of electrical noise. In the case of the HFCT sensor used, being a commercial sensor already tested in industrial environments, its behavior was more robust, and it was consequently less susceptible to external noise.

As in the previous experiments, for the data provided by the three sensors and when using the parameters *E_b_* − *ω_c_* in the visualization of the clusters (Figure 10a–c (right)), characterization of PD sources and electrical noise was not achieved.

## 6. Conclusions

This article presents a comparison of three inductive sensors and their ability to identify and separate different types of partial discharge using the chromatic technique. The separation of electrical noise from PD signals and the classification of PD sources is the paramount importance for diagnostics of electric power equipment and efficient asset management.

In general, the chromatic technique has demonstrated great potential for clustering and separating sources of partial discharges and electrical noise captured by the HFCT, ILS, and RC sensors in controlled environments and test objects, where noise sources were limited by shielding cages. When more practical/industrial test environments and test objects were deployed, and the signal acquisition included simultaneous PD sources, the ILS and RC sensors provided signals that were more difficult to separate using the chromatic technique. The authors believe that, by improving the design and construction of these sensors, the SNR and the results of the separation technique will be also improved. For example, the ILS sensor could be shielded to be able to only capture signals at the point where the sensor is coupled.

In most cases, the HFCT proved to be a more robust sensor against external disturbances, so it was possible to obtain better characterization of the sources in both controlled and practical environments, which evidences that this technique can be used to analyze the signals measured by this type of inductive sensor.

When the three parameters (*E_b_ ω_c_*, and *B*) used in the chromatic technique for the different types of sources of PD were evaluated individually, it was found that the parameter *E_b_* did not provide additional information in the characterization of this sources. In the case of internal and surface PD, this parameter generated high dispersion, which caused the cluster to be scattered on the *E_b_* axis of the separation map. For this reason, the alternative of using the other two parameters (*ω_c_* and *B*) in a 2D separation map was exploited, obtaining better results identifying the presence of different sources of PD and electrical noise.

Although the *E_b_* parameter was not effective in the classification process, the results cannot be considered definitive; additional tests on other test objects are required in order to obtain more conclusive results. In these additional tests, an acquisition system that allows simultaneous measurement of the three sensors could be used and thus facilitate the measuring process and improve the results comparison. Overall, the chromatic technique used here contributes to the separation of noise from PD signals and to the classification of PD sources.

## Figures and Tables

**Table 1:** Trigger levels to characterize electrical noise.

Sensor	Trigger Level (V)
HFCT	0.0021
ILS	0.0016
RC	0.0012

**Table 2:** Trigger and voltage levels to characterize PD.

SETUP	Voltage Level (kV)	Trigger Level (V) HFCT	Trigger Level (V) ILS	Trigger Level (V) RC
Point–plane setup	9	0.0134	0.0050	0.0038
Ceramic bushing	13.5			
Sheets of NOMEX	8.8			
